# The Secretaryship of St. Mark's

**Published:** 1914-08-01

**Authors:** J. P. Lockhart Mummery

**Affiliations:** Senior Surgeon, St. Mark's Hospital. 9 Hyde Park Place, W.


*To the Editor of* The Hospital.

### The Secretaryship of St. Mark's.

*To the Editor of* The Hospital.

Sir,—I noticed in your issue of July 11, under the heading " The Secretaryship of St. Mark's Hospital, City Road," an article which seems to me to suggest that there is something not quite straightforward about the manner in which St. Mark's Hospital advertised for a new secretary.

This article appears to have been inserted without any proper inquiries having been made. There was nothing in the least mysterious or unusual about the method of advertising the post. It is a rule of the hospital that all vacancies of this description must be advertised. The late secretary, Mr. A. W. Sowden, had been obliged to resign, owing to continued ill-health. The post was advertised in the usual way, and the emoluments attaching to it could have been ascertained by inquiry at the hospital. There were a large number of applicants, whose applications were most carefully considered by the board of management, and the post has now been filled by the appointment of Mr. H. W. G. Coope.

J. P. Lockhart Mummery, F.R.C.S.,
Senior Surgeon, St. Mark's Hospital.

9 Hyde Park Place, W.,
July 22, 1914.

[We are not quite clear whether Mr. Mummery is expressing his own personal views in this letter, or whether he represents the medical staff of St. Mark's Hospital, or whether he represents the board of management. In any event, the answer is perfectly simple. Noticing the advertisement in question, we made a telephonic inquiry at the hospital as to the vacancy, with the not unnatural object of publishing any information received as an item of interest to institutional workers. The inquiry was expressly stated to be made by the Editor of The Hospital, but only the most vague and mysterious answer could be elicited. As we stated in the paragraph to which Mr. Mummery objects, we were informed that Mr. Sowden was no longer in attendance at the hospital, but could get absolutely no information as to the reason for his absence. We regret to hear that ill-health had caused him to resign, but cannot understand why any mystery should have been made about so ordinary, if regrettable, a cause for resignation. We accept gladly Mr. Mummery's statement that there has been nothing mysterious or unusual about the procedure, but would ask him to note that it was the action of those in charge at the hospital itself which created the contrary impression.—Ed. The Hospital.]

### The Royal Portsmouth Hospital.

*To the Editor of* The Hospital.

